# MicroRNA-155-IFN-γ Feedback Loop in CD4^+^T Cells of Erosive type Oral Lichen Planus

**DOI:** 10.1038/srep16935

**Published:** 2015-11-23

**Authors:** Jing-Yu Hu, Jing Zhang, Jing-Zhi Ma, Xue-Yi Liang, Guan-Ying Chen, Rui Lu, Ge-Fei Du, Gang Zhou

**Affiliations:** 1 The State Key Laboratory Breeding Base of Basic Science of Stomatology (Hubei-MOST) & Key Laboratory of Oral Biomedicine Ministry of Education, School and Hospital of Stomatology, Wuhan University, Wuhan, China; 2 Department of Oral Medicine, School and Hospital of Stomatology, Wuhan University, Wuhan, P.R. China; 3 Department of Stomatology, Tongji Hospital, Tongji Medical College, Huazhong University of Science and Technology, Wuhan, P.R. China

## Abstract

Oral lichen planus (OLP) is a T cell-mediated immune disorder, and we have indicated a Th1-dominated immune response in OLP. MicroRNA-155 (miR-155) could promote Th1 cells polarization. The present study aims to determine the role of miR-155 in immune response of OLP. The expression of miR-155 and the target mRNA was tested by Real-Time PCR. The serum levels of IL-2, 4, 10 and IFN-γ were examined with ELISA. Furthermore, *in vitro* study was built to observe the function of miR-155 in erosive-type OLP (EOLP). Finally, we determined the expression and correlation of miR-155 and SOCS1 in EOLP CD4^+^ T cells. The results showed miR-155 was high related with the disease severities. Besides, serum IFN-γ was specifically increased in EOLP group, while IL-4 was decreased. *In vitro* studies showed miR-155 could reinforce IFN-γ signal transducer, and the induction of IFN-γ could also promote miR-155 expression in EOLP CD4^+^ T cells. In addition, miR-155 levels were negatively related with SOCS1 mRNA expression in EOLP CD4^+^ T cells. Our study revealed a positive miR-155- IFN-γ feedback loop in EOLP CD4^+^ T cell, which might contribute to the Th1-dominated immune response. Furthermore, miR-155 could be used for the evaluation and treatment of OLP.

Oral lichen planus (OLP) is one of the most common diseases of oral mucosa, which has been classified as a precancerous lesion by the World Health Organization (WHO)^1^,^2^,^3^. There are six recognized oral manifestations of OLP, i.e. reticular, papular, plaque, atrophic, erosive and bullous lesions, and the erosive type is considered as the most possible premalignant character of OLP^3^,^4^. So far, the exact pathogenesis of OLP remains elusive, however, many researchers supported that CD4^+^ T cells were protagonists of the immune response in OLP^5^,^6^,^7^.

The most important function of CD4^+^ T cells was producing a large number of various cytokines, in which, interferon-gamma (IFN-γ) acting via signal transducer and activator of transcription 1 (STAT1) is the key initiator for specification and cell fate commitment for T helper 1 (Th1) cells^8^. Activation of Janus kinases (JAKs) and STAT1 signaling induces the transcription factor T-bet, a master regulator that promotes Th1 cells differentiation^9^. By JAKs-SATAT1 signaling, IFN-γ could inhibit production of anti-inflammatory cytokines like IL-4 and IL-10, while promote secretion of proinflammatory cytokines like IL-2^10^,^11^. Our previous study has implicated a predominant role of Th1-type immune response in peripheral blood of OLP^5^,^6^,^7^. Meanwhile, in circulating of OLP patients, Th1 cell-related cytokines form a special cytokines environment which can be reinforced or attenuated by the epigenetic modifications^12^,^13^.

MicroRNAs (miRNAs) are 18- to 25-nucleotide (nt) single-stranded molecules that control nearly 1/3 post-transcriptional gene expression in a epigenetic way^14^. Recently, it has become evident that deregulation of mRNAs induced by miRNAs may affect human immune response, resulting in many pathogenic disorders^14^,^15^,^16^,^17^. MiR-155 is encoded within an exon of the non-coding RNA known as B cell integration cluster (Bic) gene^17^. In many immune diseases such as multiple sclerosis, rheumatoid arthritis, systemic lupus erythematosus and inflammatory bowel disease, miR-155 was found to have abnormal expression in peripheral blood of the patients^18^,^19^,^20^. It has been demonstrated that miR-155 greatly involved in the immune mechanism mediated by CD4^+^ T cells. For instance, miR-155 in activated CD4^+^ T cells could promote Th17 cell differentiation, and knocking out of bic gene might lead to a break of Th1/Th2 balance in CD4^+^ T cells^19^,^20^,^21^,^22^. Suppressor of cytokine signaling 1 (SOCS1) was considered as a key target of miR-155 in Th1 cells, which negatively regulated JAKs-SATAT1 signaling. SOCS1 was also the inhibitor of the signal transduction of certain cytokines like IFN-γ and IL-2. In addition, SOCS1 was found to have effects on the differentiation, maturation and function of CD4^+^ T cells^23^,^24^,^25^.

Herein, our aim was to determine the expression of miR-155 in peripheral blood of OLP patients, and analyze the relationship of miR-155 with the cytokines. Furthermore, through regulating miR-155 expression, observations *in vitro* were built to examine their effects on OLP CD4^+^ T cells proliferation and the levels of cytokines. Finally, certain target of miR-155 would be predicted and confirmed.

## Results

### The levels of miR-155 and cytokines in peripheral blood of OLP

**Figure 1:**
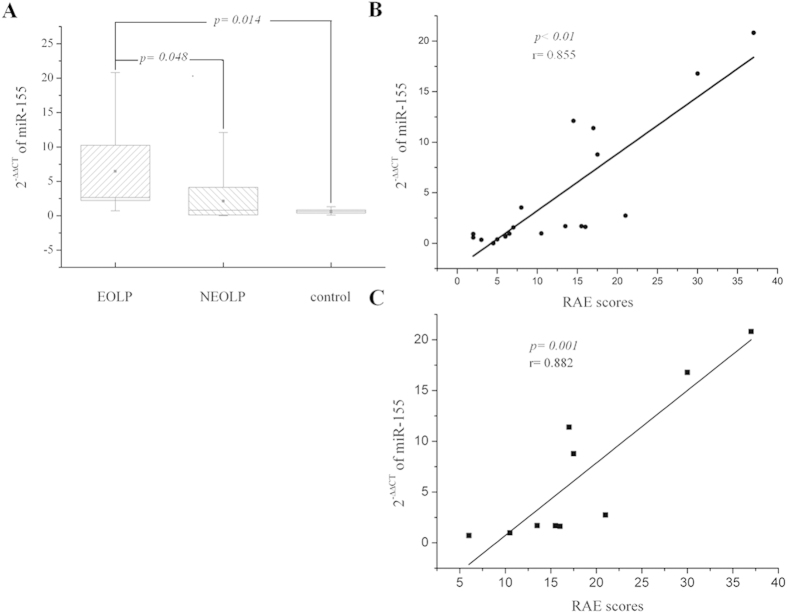
The expression of miR-155 in peripheral blood of OLP. 2^−ΔΔCT^ was used to calculate the relative expression of miR-155. The significant differences of miR-155 expression between the groups were tested by Student-Newman-Keuls (SNK) test, and results are represented as box plots. The boxes stretch from the 25th to the 75th percentile; the lines across the boxes indicate the median values; the soft dots in the box indicate the mean values; the lines stretching from the boxes indicate extreme values. Statistical significances are shown in the middle of the lines linking the two compared groups. Pearson’s correlation test was performed, and statistical significance as well as the correlation coefficient is shown. Each dot plot represents a subject, and the correlation is fitted into a straight line.

The expression of miR-155 increased in peripheral blood of EOLP patients compared with the control (*p* < 0.05), in addition, the expression of miR-155 in EOLP group was significant higher than that in NEOLP group (*p* < 0.05) (Fig. 1A). However, no difference was found between NEOLP group and the controls. Furthermore, the correlation analysis revealed that the miR-155 expression was highly related with the RAE scores which represented the severities of OLP (*p* < 0.01, r = 0.855) (Fig. 1B), and the correlation coefficient was much higher in EOLP patients (*p* < 0.01, r = 0.882) (Fig. 1C).

**Figure 2:**
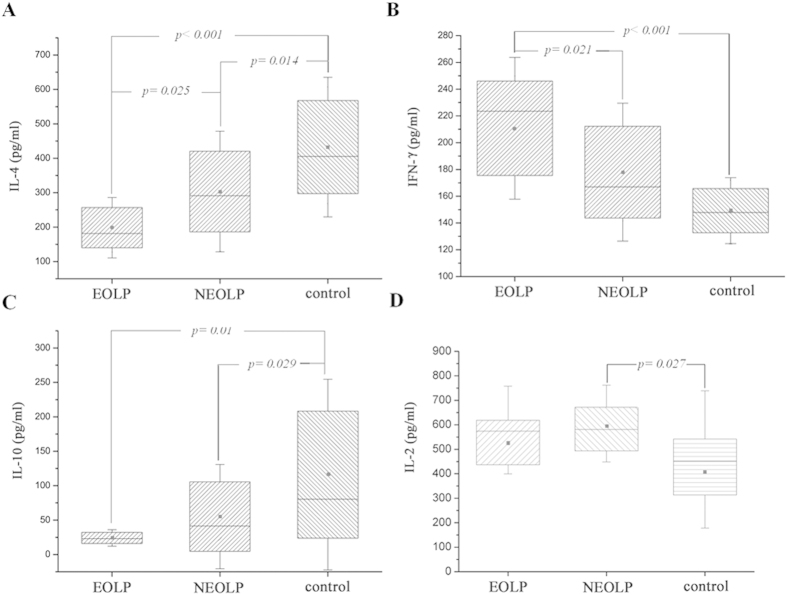
The serum levels of IL-2, IFN-γ, IL-4 and IL-10 in OLP. Kolmogorov-Smirnov test was used to confirm the data to be normal distribution. The significant differences of each cytokine levels between the groups were tested by Student-Newman-Keuls (SNK) test, and results are represented as box plots. Statistical significances are shown in the middle of the lines linking the two compared groups.

The cytokines profiles in the serum of OLP patients showed that just like the expression pattern of miR-155, the IFN-γ levels of EOLP group increased (*p* < 0.01), and were higher than that of NEOLP group (*p* < 0.05) (Fig. 2B). On the contrary, the IL-4 levels decreased in EOLP group (*p* < 0.01), and were much lower than that of NEOLP group (*p* < 0.05) (Fig. 2A). Moreover, there was descend of IL-4 levels in NEOLP group compared with the control (*p* < 0.05) (Fig. 2A). For the other two cytokines, the levels of IL-2 only increased in NEOLP group (*p* < 0.05) (Fig. 2D), IL-10 levels in the two OLP groups both declined (*p* < 0.05, *p* = 0.01), and there was no difference between them (Fig. 2C).

### The impacts of miR-155 regulation on CD4^+^ T cell proliferation as well as the levels of IFN-γ and IL-4 in the supernatant

**Figure 3:**
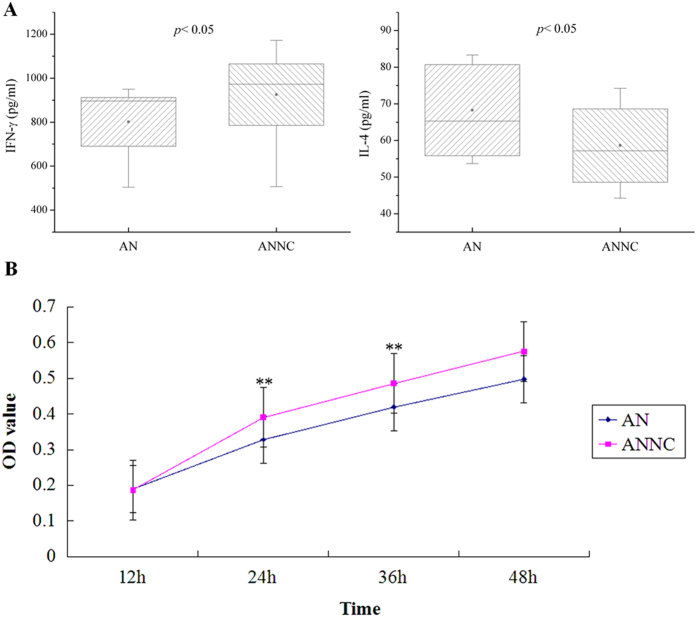
The proliferation activity as well as the IFN-γ and IL-4 levels in supernatant of EOLP CD4^+^ T cells transfected with antagomir-155. (**A**) The significant differences of IFN-γ and IL-4 levels were tested by independent-samples *t* test, and results are represented as box plots. AN represented the antagomir-155 transfected group, and ANNC was the corresponding negative controls. Statistical significances are shown in the blanks. (**B**) The solid dots and or cube separately showed the mean value of proliferation activity of EOLP CD4^+^ T cells in two groups at different time point, and the lines stretching from the dot or cube indicated extreme values. For each time point, independent-samples *t* test was used to determine differences between the two groups, in addition, “*”, “**” represent *p* < 0.05 and *p* < 0.01, respectively.

**Figure 4:**
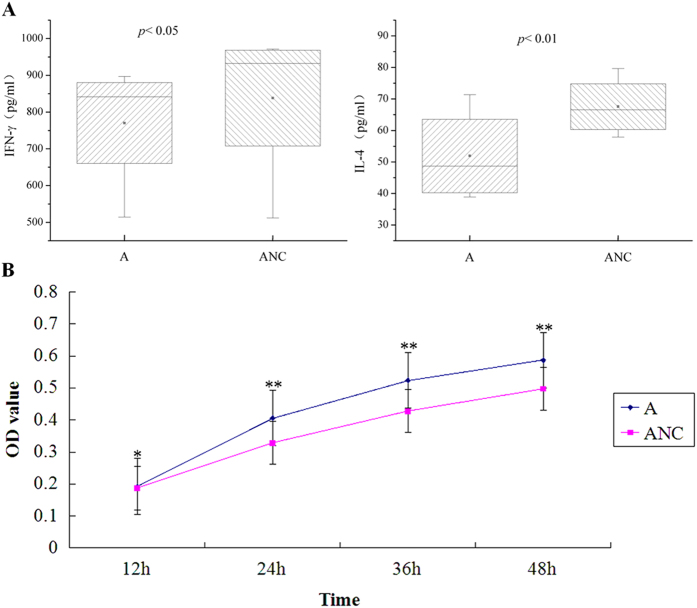
The proliferation activity as well as the IFN-γ and IL-4 levels in supernatant of EOLP CD4^+^ T cells transfected with agomir-155 (the details was illustrated as Fig. 3).

As shown in Fig. 3, when miR-155 was inhibited by antagomir-155, the proliferation of EOLP CD4^+^ T cell was declined at the time point of 24 h (*p* < 0.01) and 36 h (*p* < 0.01) compared with the negative control group (ANNC group). In addition, the levels of IFN-γ were decreased in the supernatant (*p* < 0.05), but the levels of IL-4 were increased (*p* < 0.05). when miR-155 was promoted by agomir-155, the proliferation of EOLP CD4^+^ T cell was raised at all the four time point (12 h, *p* < 0.05; 24 h, 36 h, 48 h, *p* < 0.01) compared with the negative control group (ANC group). Moreover, the levels of IFN-γ and IL-4 in the supernatant both decreased (IFN-γ, *p* < 0.05; IL-4, *p* < 0.01) (Fig. 4).

### The levels of miR-155 in EOLP CD4^+^ T cell induced with IFN-γ

**Figure 5:**
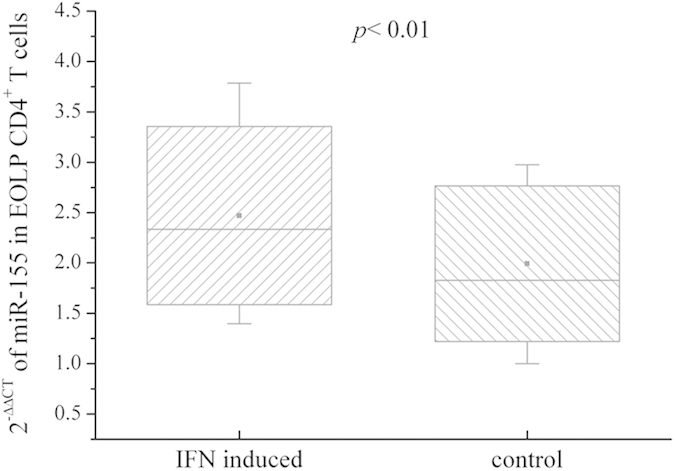
The expression of miR-155 in EOLP CD4^+^ T cells induced with IFN-γ. The significant differences between IFN-γ induced group and the blank controls were tested by independent-samples t test, and results are represented as box plots.

After induced by IFN-γ for 24 h, the expression of miR-155 in EOLP CD4^+^ T cell was increased compared with the blank control group (Fig. 5).

### The expression of miR-155 and SOCS1 mRNA in EOLP CD4^+^ T cell

**Figure 6:**
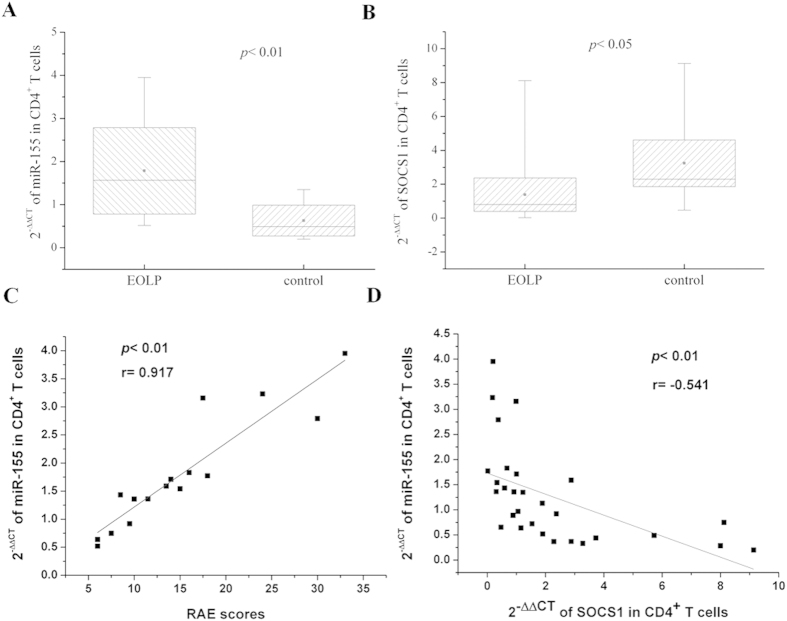
The expression of miR-155 and SOCS1 mRNA in CD4^+^ T cells from EOLP patients. (**A,B**) 2^−ΔΔCT^ was used to calculate the relative expression of miR-155 and SOCS1 mRNA. The significant differences between EOLP group and the healthy controls were tested by independent-samples t test, and results are represented as box plots. (**C,D**) Pearson’s correlation test was performed, and statistical significance as well as the correlation coefficient is shown. Each dot plot represents a subject, and the correlation is fitted into a straight line.

After browsing the potential targets, SOCS1 was found to have one and only one conserved site combined with miR-155. For the first batch of samples, the mean value of SOCS1 mRNA expression in EOLP CD4^+^ T cell was higher than that of healthy controls, but there was no statistical difference between them (data not shown). Owing to the insufficient of samples, we supplemented the sample number of both EOLP group and the control group. As shown in Fig. 6A,B, it demonstrated that in EOLP group, the expression of miR-155 of CD4^+^ T cell was increased (*p* < 0.01), but the expression of SOCS1 mRNA was decreased (*p* < 0.05). Furthermore, the correlation analysis revealed that the expression of miR-155 in CD4^+^ T cell was high related with the RAE scores of EOLP patients (*p* < 0.01), and the correlation coefficient reached up to 0.917 (Fig. 6C). In addition, it demonstrated a negative correlation between the SOCS1 mRNA expression and the miR-155 levels (*p* < 0.01, r = −0.541) (Fig. 6D).

## Discussion

The miRNAs in peripheral blood were easy to be collected and detected, thus, many researches aimed to find disease-related miRNAs in circulation to assist the diagnosis and study the remote and systemic regulation of miRNA^16^,^17^,^26^,^27^,^28^. Through screening the sera, Nylander E had ever drawn a pessimistic prediction that there was no specific LP-associated miRNA profile in peripheral blood^29^. However, in 2013, our group first reported that miR-125a was down-regulated in peripheral blood of OLP patients^5^. In the present study, the result showed that miR-155 was up-regulated in peripheral blood of EOLP patients. Furthermore, miR-155 was found to be high related to the RAE scores of OLP patients (*p* < 0.01, *r* = 0.855), and the relationship coefficient was higher when the subjects was limited to EOLP patients (*p* < 0.01, *r* = 0.882). It implicated that miR-155 might be a positive candidate to assess the severities of EOLP patients.

We and some researchers had proved that there was chasm about the immune mechanisms of EOLP and NEOLP in peripheral blood, like the expression of transcription factor, the activation of T cells and the levels of cytokines. Furthermore, clinically, these two types of OLP showed great difference in treatment, outcome and the malignant risk^3^,^5^,^6^,^7^. As the inflammatory bowel disease had two predominant types of crohn’s disease and ulcerative colitis, in our opinion, NEOLP and EOLP might be considered as two types of diseases but not just different manifestations of OLP^30^. The current data represented that the expression of miR-155 in EOLP group was higher than in NEOLP group; likewise, the level of IFN-γ increased in EOLP group, and was elevated compared with NEOLP group. But nearly the opposite to miR-155 or IFN-γ, the IL-4 level decreased in EOLP group, and was much lower than that of NEOLP group. The serum levels of IL-2 and IL-10 were distinguished between OLP groups with the controls, but no distinction was presented between EOLP group and NEOLP group. Our data further proved that NEOLP and EOLP were quite different in cytokine levels and miRNA expression, and that the immune response of EOLP tended to be much more Th1-dominated than that of NEOLP. In addition, it also revealed that the expression of miR-155 in peripheral blood might be associated with the levels of IFN-γ and IL-4.

IFN-γ could promote Th1 cell differentiation by induction of T-bet, a transcription factor critical to Th1 cell differentiation, and Th1 cells could produce IFN-γ, which formed a positive feedback to reinforce Th1-dominated immune response^10^,^11^. A second way in which IFN-γ was thought to contribute to Th1 immune response functioned by inhibiting the proliferation of Th2 as well as the secretion of IL-4^31^. IL-4 could play the same role in Th2-dominated immune response. Both IFN-γ and IL-4 should exert their function by binding to their receptors on the membrane and being assisted by JAKs/STAT pathway (JAKs/STAT1 for IFN-γ and JAKs/STAT6 for IL-4)^32^. The receptor of IFN-γ (IFN-γR) had two chains, IFN-γRα and IFN-γRβ, in which IFN-γRα was responsible for the combination with IFN-γ, while IFN-γRβ was related to the process of signal transduction, in addition, committed Th1 cells could regulate the expression of IFN-γRβ and help CD4^+^ T cells to resist the anti-proliferation effect of IFN-γ^31^,^33^,^34^. In most references, miR-155 was reported to inhibit the Th2 cell differentiation and the production of IL-4 by targeting c-MAF, a distinct transcription factor of Th2 cells which was crucial for IL-4 gene transcription^33^. In addition, miR-155 could enhance Th1 cell differentiation by targeting SOCS1, an inhibitor of JAKs/STAT1 pathway, and strengthen the signal transduction of IFN-γ^22^,^35^. However, there was still debating like Banerjee *et al.* announcing that miR-155 could target IFN-γRα to inhibit IFN-γ signal transduction^31^.

The current study showed that in the supernatant, IFN-γ levels decreased while IL-4 levels increased in the presence of antagomir-155, and the proliferation activity of EOLP CD4^+^ T cells was abated at 24 h and 36 h post-transfection. This part seemingly agree on the mainstream attitude, as miR-155 was suppressed, the pent-up signal transduction of IFN-γ could not lead to Th1 cell differentiation, and the production of IFN-γ was decreased either. On the contrary, the weakening signal transduction of IFN-γ enhanced the IL-4 secretion and Th2 differentiation. The past work had demonstrated a Th1-dominated immune response in EOLP CD4^+^ T cells, and this may be why the proliferation activity was decreased at 24 h and 36 h. In the early 12 h, for the compensation of attenuated IFN-γ signal transduction, there was less effect on anti-proliferation of IFN-γ, thus, the proliferation activity showed no difference with the negative control group. However, for the last 12 h (36–48 h), the increasing Th2 cells gradually made up for the decreasing differentiation of Th1 cells, and the immune condition might have been changed.

In the presence of agomir-155, IL-4 levels decreased in the supernatant, and the proliferation activity of EOLP CD4^+^ T cells was strengthened in the whole process. It could be explained by the enhancement of IFN-γ signal transduction regulated by miR-155, which promoted the Th1 cell differentiation as well as the declination of IL-4 production. For the effect of IFN-γ on anti-proliferation, the proliferation activity of EOLP CD4^+^ T cells did not show at first timing point (*p* < 0.05) significant difference as that of the following three timing point (*p* < 0.01), when the effect was inhibited by regulating the expression of IFN-γRβ. However, it seemed hard to elaborate why the levels of IFN-γ in supernatant was decreased. A speculation was made that the growing EOLP CD4^+^ T cells recruited more and more IFN-γ combining with the IFN-γRα on the membrane to keep the enhancement of IFN-γ signal transduction, and the dissociated IFN-γ in the supernatant declined compared with the negative control group. Furthermore, we found that the induction of IFN-γ could also promote the expression of miR-155 in EOLP CD4^+^ T cells. All these results might reveal a positive feedback of IFN-γ signal transduction and miR-155 expression in EOLP CD4^+^ T cells: when miR-155 is over-expressed, the signal transduction of IFN-γ is unlimited, which activates the bic gene, and promotes the miR-155 expression. Same phenomenon was observed in innate immune response, but there was no explicit mechanism^36^,^37^. The feedback of IFN-γ and miR-155 might play an important role in the Th1-dominated immune response of EOLP.

The most possible target of miR-155 to promote the IFN-γ signal transduction was SOCS1, which had one and only one conservative binding sites with miR-155 (Targetscan)^22^,^31^,^35^,^38^. The data showed SOCS1 mRNA decreased in EOLP CD4^+^ T cells (*p* < 0, 05), and there was a clear negative correlation between SOCS1 mRNA and miR-155 expression (*p* < 0.01, r = −0.541). These results might prove that through targeting SOCS1, miR-155 and IFN-γ formed a positive feedback in EOLP CD4^+^ T cells. By the way, the expression of miR-155 in EOLP CD4^+^ T cells represented an extremely high positive relationship with the RAE scores of EOLP patients (*p* < 0.01, r = 0.917).

In conclusion, we found miR-155 was highly expressed in the peripheral blood of EOLP patients, and was high related with the severity of OLP. Furthermore, a positive feedback loop of miR-155 and IFN-γ was found in EOLP CD4^+^ T cells, which might contribute of the Th1-dominated immune response in EOLP, and SOCS1 was considered to be the most possible target of miR-155 involved in the feedback loop.

## Materials and Methods

This experiments followed the principles outlined in the Declaration of Helsinki in the use of human samples and were approved by the Ethics Committee of School and Hospital of Stomatology, Wuhan University with approved NO 2011051. All participating subjects gave their informed consents.

### Patients and controls

The patients involved in this study were clinically and pathologically diagnosed as OLP according to the definition of OLP made by the WHO^4^. In term of the manifestation, they were divided into two groups: erosive type OLP (EOLP) group and non-erosive type OLP (NEOLP) group^39^. Age and gender matched healthy volunteers were recruited as the controls. In the first stage, 10 EOLP patients, 10 NEOLP patients and 10 controls was recruited, and in the second stage, 7 more EOLP patients and 3 more controls were replenished. Table 1 displays the clinical details. The subjects neither had any systemic disorders (such as cardiovascular disease, diabetes mellitus, etc) nor any soft tissue lesions in the oral mucosa. Smokers and severe alcoholics were excluded. Besides, patients on immunotherapy, receiving any medical treatment of OLP (local or systematic) within 3 months or having medicines affecting RNA synthesis and transcription in 6 months should not be included. All patients recruited in this study had been treated as needed following the sample collection.

### Evaluation of the severities of OLP patients

RAE (reticular, atrophic and erosive) scoring system recommended by our previous study, which has shown much practicality and efficiency, was used to assess the severity of OLP in different clinical forms (Table 2)^5^,^6^.

### Serum and EOLP CD4^+^ T cell isolation

Fourteen milliliter peripheral blood sample was drawn from each subject. Two milliliter blood (without anticoagulation) was kept in room temperature for 1 h, and then centrifuged at 3000rpm for 10 min, and stored the supernatant serum in −20 °C. Another ten milliliter blood (with anticoagulation) was diluted with equivalent phosphate buffers (PBS) and the sample was transferred to the centrifuge tubes with lymphocytes separation medium (tbdscience Biotech Ltd, Tianjin, China). Isolation of peripheral blood mononuclear cells from EOLP patients was performed by Ficoll-Paque density gradient centrifugation, and then a human CD4 T lymphocyte enrichment set-DM (BD IMag^TM^ Becton, Dickinson and Company, NJ, USA) was applied for negative selection of CD4 T lymphocyte. The isolated CD4^+^ T cells were maintained with RPMI 1640 culture medium containing 20% fetal bovine serum (Gibco® Life Technologies, CA, USA) at 2 × 105 cells/ml.

### IFN-γ inducement and miR-155 regulation in EOLP CD4^+^ T cell

Recombinant Human IFN-γ (PeproTech, NJ, USA) was reconstituted in water to a concentration of 1.0 mg/ml. Agomir-155 (a modified RNA oligomer specifically increase miR-155 activity), antagomir-155 (a modified antisense RNA oligomer specifically reduce miR-155 activity) and their negative control reagents were prepared as 20 μmol/L working solution (Shanghai GenePharma Co.,Ltd, Shanghai, China). For each sample, added 2 ml EOLP CD4^+^ T cells cultured solution (cell viability > 95%) to two wells of 12-well plate to make sure each well containing 2 × 10^5^ CD4^+^ T cells, meanwhile, sixteen wells in 96-well plate were filled with 6000 CD4^+^ T cells, and expanded the volume to 200 μl with Opti-MEM® I Reduced-Serum Medium (Gibco® Life Technologies, CA, USA). Then 10 ng Recombinant Human IFN-γ was appended into one well with CD4^+^ T cells in 12-well plate, and agomir-155, antagomir-155 as well as their negative control regents were transfected into the CD4^+^ T cells of the sixteen wells in 96-well plate with proper Lipofectamine 2000 Reagent (Life Technologies, CA, USA) to make sure that the concentration of agomir-155 and its negative control regents was 50 nM, and for antagomir-155 and its negative control regents, the concentration was 100 nM. The IFN-γ induced CD4^+^ T cells were marked as IFN-γ induced group, and CD4^+^ T cells transfected with agomir-155, antagomir-155 and their negative control regents were marked as A group, AN group, ANC group, ANNC group, respectively. After 24 h, IFN-γ induced CD4^+^ T cells as well as the supernatants from A group, AN group, ANC group and ANNC group were collected.

### Proliferation assay of EOLP CD4^+^ T cells

Cell counting kit-8 (CCK8) was applied to estimate the proliferation of CD4^+^ T cells (KeyGEN BioTECH Co.,Ltd, Nanjing, China). For each sample, two wells filled with CD4^+^ T cells in 96-plate from A group, AN group, ANC group, ANNC group, respectively were taken. Then added 20 μl WST-8 to each well, and build a consecutive observation with the time point of 12 hour, 24 hour, 36 hour and 48 hour. At each timing point, the OD value of each well was rescored using Synergy 2 Multi-Mode Reader (BioTekInstruments, Inc, VT, USA).

### RNA extraction and reverse transcription

Total RNA was extracted from peripheral blood (the last two milliliter with anticoagulation) using miRNeasy Mini Kit (QIAGEN GmbH, Düsseldorf, Germany). Likewise, the RNA of EOLP CD4^+^ T cells were extracted with miRNeasy Micro Kit (QIAGEN GmbH, Düsseldorf, Germany). Concentration and purity were then determined by NanoDrop 1000 spectrophotometer (Thermo Fisher Scientific Inc, Dubuque, USA). cDNA was synthesized from the total RNA using miScript Reverse Transcription Kit (QIAGEN GmbH, Düsseldorf, Germany). The conditions were under 60 min at 37 °C and 5 min at 95 °C.

### Quantitative real-time polymerase chain reaction (Real-Time PCR) analysis for miR-155 and SOCS1 mRNA

The analyses of miR-155 expression were done with miScript SYBR^®^ Green PCR Kit (QIAGEN GmbH, Hilden, Germany) using ABI 7500 Real-Time PCR system (Applied Bio-systems Inc, Foster City, CA, USA) in a one-step real-time PCR 96-well optical plate. The PCR initial activation step was at 95 °C for 15  min, followed by 15 s at 94 °C for denaturation, 30 s at 55 °C for annealing, and 34 s at 70 °C for extension. The cycle number was set as 35 cycles. MiR-155 expression was normalized to endogenous miRNA-U6 as internal control. The forward primer and reverse primer of U6 were synthesized as CTCGCTTCGGCAGCACA and AACGCTTCACGAATTTGCGT, respectively (Invitrogen ™ Life Technologies, Shanghai, China). The forward primer sequence of miR-155 was designed as TTAAT GCTAA TCGTG ATAGGG (Invitrogen ™ Life Technologies, Shanghai, China), and a miscript universal primer had already been offered in the PCR kit. The analyses of SOCS1 mRNA expression in EOLP CD4^+^ T cells were determined with FastStart Universal SYBR Green Master (ROX) (Roche Applied Science, Bael, Swiss) according to the manufacturer’s instructions. SOCS1 mRNA expression was normalized to endogenous hGAPDH mRNA as internal control. The forward primer of SOCS1 and hGAPDH mRNA were synthesized as GCCCTTAGCGTGAAGATGG and CTTTGGTATCGTGGAAGGACTC, respectively, in addition, the reverse primer were synthesized as TGTGCGGAAGTGCGTGT and GTAGAGGCAGGGATGATGTTCT, respectively (Invitrogen™ Life Technologies, Shanghai, China). The threshold cycle (Ct) of three replicates and two replicates for internal control per sample was used to calculate 2^−ΔΔCT^.

### Enzyme-linked immunosorbent assay (ELISA)

ELISA kit (R&D Systems, Minneapolis, MN, USA) was used to test the levels of interleukin 2 (IL-2), IL-4, IL-10 and IFN-γ in serum as well as the supernatant of CD4^+^ T cells according to the manufacturer’s instructions.

### Statistical analyses

Data were presented as mean ± SD, and statistical significance was defined as *p* value < 0.05. The One-Way ANOVA and Pearson’s correlation test were performed by SPSS 13.0 for windows software (SPSS, Inc, Chicago, USA).

### Target prediction

Using TargetScan (version 6.2; http://www.targetscan.org), we looked up the potential targets of miR-155^40^.

## Additional Information

**How to cite this article**: Hu, J.-Y. *et al.* MicroRNA-155-IFN-γ Feedback Loop in CD4^+^T Cells of Erosive type Oral Lichen Planus. *Sci. Rep.* **5**, 16935; doi: 10.1038/srep16935 (2015).

## Figures and Tables

**Table 1:** Clinical characteristic of OLP patients and the controls.

	EOLP group	NEOLP group	Controls	added EOLP cases	added controls
Number	10	10	10	7	3
Age(year)					
Mean	44	41	49	46	48
Range	40–63	24–63	37–55	44–50	45–52
Gender					
Female	5	5	5	4	2
Male	5	5	3	3	1
Disease duration (month)					
Mean	29.1	11	27.9		
Range	2–72	3–30	22–40		

**Table 2:** The RAE scoring system for OLP.

Clinical form	Scoring
Reticular	0 = no white striations; 1 = presence of white striations or keratotic papules
Atrophic	0 = no lesion; 1 = lesions < 1 cm2; 2 = lesions from 1 to 3 cm^2^; 3 = lesions > 3 cm^2^
Erosive	0 = no lesion; 1 = lesions < 1 cm^2^; 2 = lesions from 1 to 3 cm^2^; 3 = lesions > 3 cm^2^
Total^a^	∑R + ∑ (A × 1.5) + ∑ (E × 2.0)

^a^The oral cavity was divided into 10 sites: upper/lower labial mucosa, right buccal mucosa, left buccal mucosa, dorsal tongue, ventral tongue, floor of mouth, hard palate mucosa, soft palate/tonsillar pillars, maxillary gingiva and mandibular gingival. The total score was recorded by summation of the scores of all 10 areas multiplied by a weighted factor of 1.5 (atrophic lesion) or 2.0 (erosive lesion)^5^,^6^.
